# Supplementary material for: Temporal dynamics of Sertoli and germ cell development in human foetal and prepubertal testis
Source: Biol Open. 2026 Feb 6;15(2):bio062319. doi: 10.1242/bio.062319 (PMC12919961; doi:10.1242/bio.062319)
Supplement: Supplementary information [file biolopen-15-062319-s1.pdf]

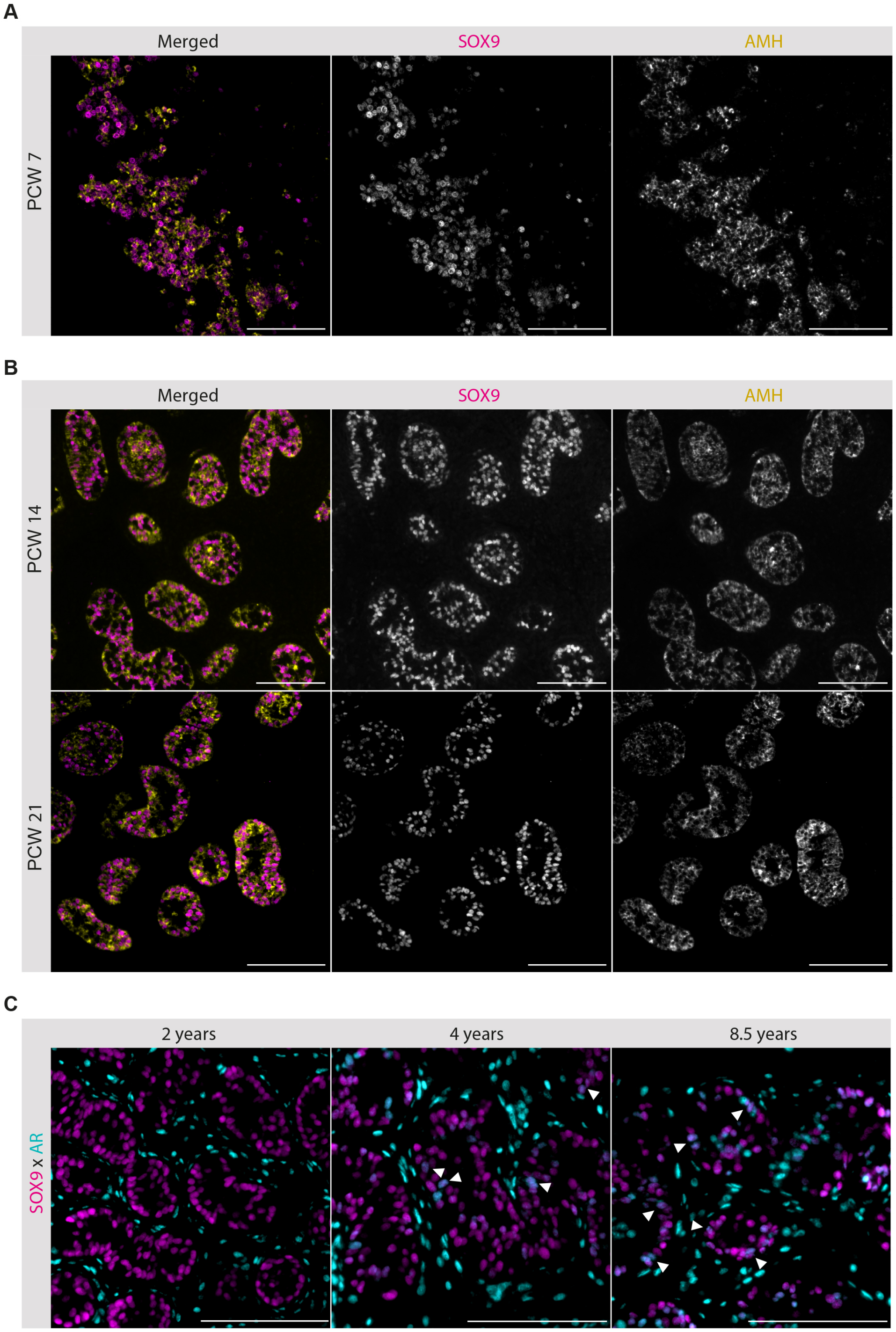

Fig. S1. Expression dynamics of key Sertoli cell maturation markers during human foetal and prepubertal testis development. A. Representative images indicating SOX9 (magenta) and AMH (yellow) co-expression in Sertoli cells in PCW 7 foetal testis. Scale bar = 100 µm. The (merged) image of PCW 7 is from Fig 2A. **B.** Representative images showing partial basal localisation of SOX9 (magenta) and AMH (yellow) at PCW 14, progressing to a complete basal localisation by PCW 21. Scale bar = 100 µm. The (merged) images of PCW 21 is from Fig. 2A. **C.** Representative images showing co-expression of SOX9 (magenta) and AR (cyan) in a 2, 4 and 8.5 year old prepubertal testis, with white arrowheads indicating SOX9 and AR double positive Sertoli cells, marking the onset and increasing prevalence of Sertoli cell maturation from 4 year onwards. Scale bar = 100 µm.

Biology Open • Supplementary information

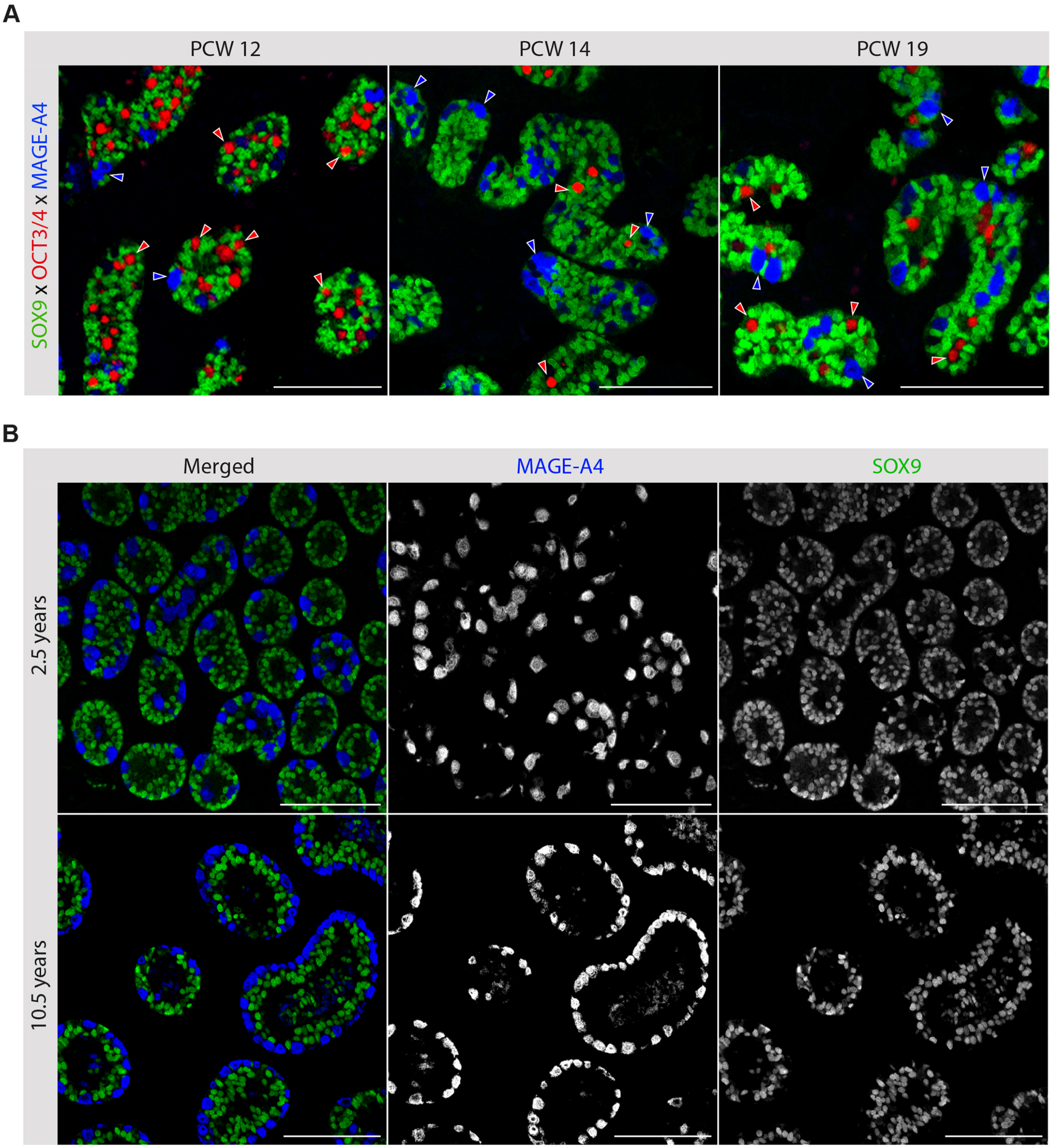
A
PCW 12
PCW 14
PCW 19
SOX9 x OCT3/4 x MAGE-A4
B
Merged
MAGE-A4
SOX9
2.5 years
10.5 years

Fig. S2. Localisation and nuclear morphology of OCT3/4+ gonocytes and MAGE-A4+ spermatogonia during human testis development. A. Representative images showing OCT3/4 (red), MAGE-A4 (blue) and SOX9 (green) expression in foetal testis at PCW 12, 14 and 19. Red arrowheads indicate OCT3/4+ gonocytes that display a smaller, round nuclei and are typically positioned towards the adluminal side of the seminiferous cords, while blue arrowheads indicate MAGE-A4+ (pre)spermatogonia that exhibit a larger nuclei and are predominantly localised at the basal region. Scale bar = 100 µm. **B.** Representative images showing MAGE-A4 (blue) and SOX9 (green) expression in prepubertal testis aged 2.5 and 10.5 years. MAGE-A4 spermatogonia are occasionally observed at the adluminal regions at 2.5 years but are consistently restricted to the basal membrane at 10.5 years. Scale bar = 100 µm. The (merged) image of 2.5 years is from Fig. 3A.

A

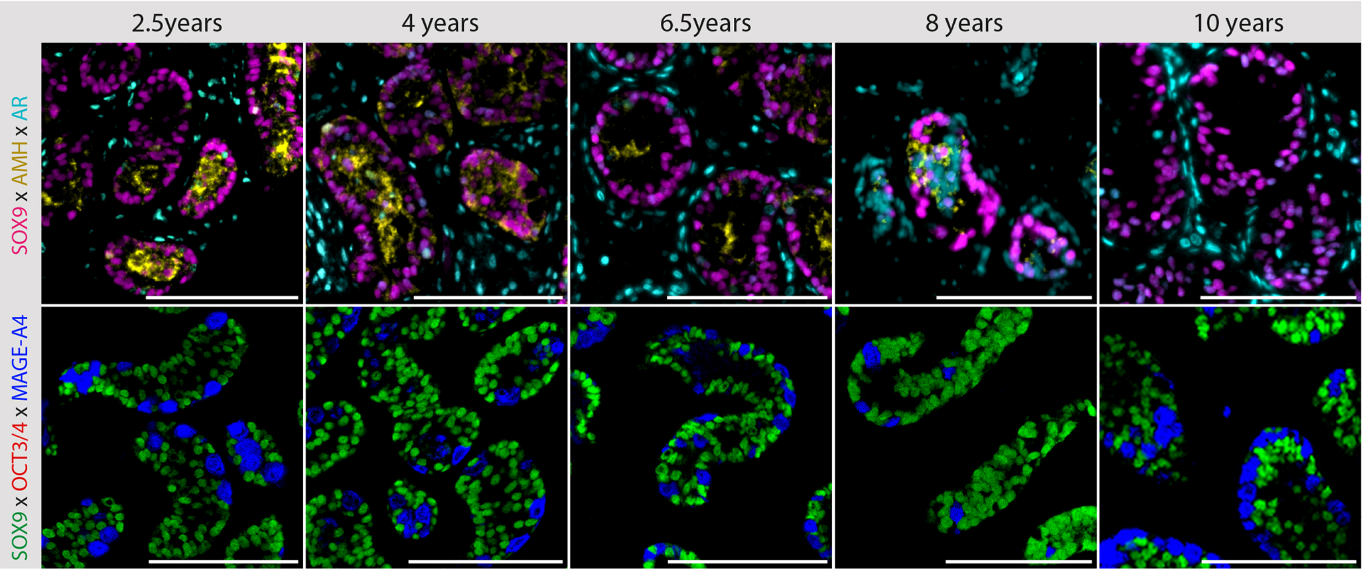

Fig. S3. Intermediate prepubertal time points illustrating Sertoli and germ cell maturation. Representative images of prepubertal testes aged 2.5, 4, 6.5, 8 and 10 years. **Top row** shows SOX9 (magenta), AMH (yellow) and AR (cyan) expression, illustrating progressive Sertoli cell maturation with age, characterised by loss of AMH expression and gradual acquisition of AR expression. **Bottom row** shows SOX9 (green) and MAGE-A4 (blue) expression, highlighting the increasing basal restriction of MAGE-A4 spermatogonia, and formation of the lumen from 6.5 years onwards. OCT3/4 (red) expression is absent in all postnatal samples, consistent with the loss of pluripotent gonocytes from early postnatal life. Scale bar = 100 µm. The (merged) images of 2.5 years and 10 years (top row) are from Fig. 2A, and the (merged) images from 2.5 years and 4 years (bottom row) are from Fig. 3A.

Table S1. Measured cord/tubular diameter (μm)

Available for download at
https://journals.biologists.com/bio/article-lookup/doi/10.1242/bio.062319#supplementary-data

Table S2. Human tissue resources used in this study

Available for download at
https://journals.biologists.com/bio/article-lookup/doi/10.1242/bio.062319#supplementary-data

Table S3. Raw counts for assesment of SOX9, AMH and AR positive tubules in sections

Available for download at
https://journals.biologists.com/bio/article-lookup/doi/10.1242/bio.062319#supplementary-data

Table S4. Descriptive statistics. Mean proportion and standard deviation (S.D.) per age used in this study. Standard deviations are not applicable for ages with n=1

Available for download at
https://journals.biologists.com/bio/article-lookup/doi/10.1242/bio.062319#supplementary-data

Table S5. Raw counts for assessment of SOX9, MAGE-A4, OCT3/4 and Ki67. ABN=absolute number

Available for download at
https://journals.biologists.com/bio/article-lookup/doi/10.1242/bio.062319#supplementary-data

Table S6. Chemicals and reagents used in this study

Available for download at
https://journals.biologists.com/bio/article-lookup/doi/10.1242/bio.062319#supplementary-data

Table S7. Antibodies used in this study

Available for download at
https://journals.biologists.com/bio/article-lookup/doi/10.1242/bio.062319#supplementary-data
